# Dynamics and Predictive Values of Urinary Podocyte Biomarkers Following SGLT2 Inhibition in CKD

**DOI:** 10.3390/life16030529

**Published:** 2026-03-23

**Authors:** Alexandra Urs, Diana Moldovan, Crina Claudia Rusu, Cosmina Ioana Bondor, Alina Ramona Potra, Dacian Tirinescu, Maria Țicală, Yuriy Maslyennikov, Andrada Alina Bărar, Ioana Dînșoreanu, Ina Maria Kacso

**Affiliations:** 1 Department of Nephrology, Iuliu Hațieganu University of Medicine and Pharmacy, 8 Victor Babeș Street, 400012 Cluj-Napoca, Romania; alexandra.urs@elearn.umfcluj.ro (A.U.); diana.moldovan@umfcluj.ro (D.M.); claudia.rusu@umfcluj.ro (C.C.R.); alina.potra@umfcluj.ro (A.R.P.); tirinescu.dacian@umfcluj.ro (D.T.); cosa.maria@umfcluj.ro (M.Ț.); maslyennikov.yuriy@umfcluj.ro (Y.M.); andrada.barar@yahoo.ro (A.A.B.); ioana.dinsoreanu@gmail.com (I.D.); maria.kacso@umfcluj.ro (I.M.K.); 2 Department of Nephrology, Emergency County Clinical Hospital Cluj-Napoca, 3-5 Clinicilor Street, 400006 Cluj-Napoca, Romania; 3 Department of Medical Informaticsand Biostatistics, Iuliu Hațieganu University of Medicine and Pharmacy, 6 Pasteur Street, 400349 Cluj-Napoca, Romania

**Keywords:** podocyte injury, SGLT2 inhibitors, chronic kidney disease, podocalyxin, podocin, nephrin, albuminuria

## Abstract

Podocyte injury is an early hallmark of chronic kidney disease (CKD) and can be influenced by SGLT2 inhibitors (SGLT2i). Early effects of SGLT2i include transient estimated glomerular filtration rate (eGFR) dip and reduced proteinuria; however, the latter may be subtle in patients with normal or moderately increased albuminuria. In such cases, urinary podocyte-derived biomarkers may provide sensitive early indicators of SGLT2i effect. This study prospectively assessed short-term changes in urinary podocyte biomarkers (nephrin, podocin, podocalyxin) following SGLT2i initiation in diabetic and non-diabetic CKD patients. Our cohort had a low urinary albumin to creatinine ratio (UACR) of 19.09 mg/g (6.28; 164.93) and preserved eGFR 45 mL/min/1.73 m^2^ (36.85; 53.15). At baseline, podocyte biomarkers were mutually correlated, whereas only podocalyxin was associated with UACR. Baseline podocalyxin independently predicted transient eGFR dip and UACR reduction. Early decreases in both podocin and podocalyxin were associated with eGFR decline and lower UACR at 3 months. ROC analyses identified cutoff values for all three biomarkers that predicted short-term eGFR decline, with baseline podocalyxin demonstrating the highest discriminative accuracy. These findings support pleiotropic nephroprotective effects of SGLT2 inhibitors and identify urinary podocyte biomarkers—particularly podocalyxin, and to a lesser extent podocin—as a sensitive indicator of early renal response.

## 1. Introduction

Sodium–glucose-cotransporter-2 inhibitors (SGLT2i) have been demonstrated to slow the progression of chronic kidney disease (CKD) and are now a cornerstone of modern CKD management. Starting with the CREDENCE trial and subsequently confirmed in DAPA-CKD and EMPA-KIDNEY [1,2,3], these agents have provided substantial renal benefits in both diabetic and non-diabetic populations.

The predominant mechanism underlying SGLT2 inhibitor–mediated renoprotection is restoration of tubulo-glomerular feedback, but multiple additional pathways have been proposed [4]. Among these, preservation of podocyte integrity has gained increasing attention. Experimental studies have shown that SGLT2 inhibition improves podocyte ultrastructure and function, likely mediated by diverse intracellular signaling mechanisms [5]. In light of the recent description of SGLT2 receptors on podocytes [6], this mechanism became even more appealing.

In human studies, direct assessment of podocyte ultrastructure is challenging; however, over the past two decades, several podocyte-derived biomarkers have been identified as sensitive indicators of early podocyte injury and imbalance. As such, urinary excretion of podocyte-associated proteins—including nephrin, podocin, and podocalyxin—can be detected early in the disease course, often preceding albuminuria, in both diabetic and non-diabetic glomerular disease settings [7,8]. It is well known that early reduction in proteinuria after SGLT2 inhibitors initiation is a key marker of therapeutic efficacy and predicts long-term renal benefit [9,10]. Therefore, the dynamics of these urinary biomarkers may be particularly valuable in non-albuminuric patients, in whom short-term proteinuric response following SGLT2 inhibition is challenging to ascertain.

Clinical studies have not extensively explored this issue. To the best of our knowledge, only five clinical studies have evaluated the impact of SGLT2 inhibitors on alleviating podocyte injury [11,12,13,14,15].

Hence, we aimed to analyze short-term changes in podocyte-derived biomarkers following initiation of SGLT2 inhibitors in a cohort of CKD patients, including both diabetics and non-diabetics, and to examine associations between these markers and renal function, assessed using estimated glomerular filtration rate (eGFR) and albuminuria.

## 2. Materials and Methods

### 2.1. Patients and Study Design

This single-center prospective study enrolled consecutive diabetic and non-diabetic SGLT2 inhibitors naïve CKD patients evaluated in the outpatient setting of the Emergency County Hospital Cluj between July 2024 and August 2025. A total of 88 patients were included in our cohort and received SGLT2 inhibitor therapy at baseline. CKD-related parameters were monitored prior to treatment initiation and reassessed at 3 months of follow-up.

### 2.2. Study Subjects and Inclusion and Exclusion Criteria

Patients were eligible for inclusion if they had a confirmed diagnosis of CKD (the existence of KDIGO criteria was confirmed upon inclusion in the study, based on laboratory reports), with evidence of persistent renal impairment for more than three months, defined by either elevated albuminuria or a reduced eGFR. Participants had to be naïve to SGLT2i therapy and have a clinical indication for SGLT2 initiation according to the national protocol [16]. This included patients with an eGFR between 25 and 60 mL/min/1.73 m^2^ regardless of albuminuria, those with an eGFR of 60–75 mL/min/1.73 m^2^ in the presence of albuminuria ≥30 mg, or patients with eGFR >75 mL/min/1.73 m^2^ and albuminuria between 30 and 200 mg. All participants were required to be willing to comply with study procedures and to provide informed consent.

Exclusion criteria comprised age under 18 years, pregnancy or lactation, and known hypersensitivity or contraindications to SGLT2i. Patients with serious concomitant medical conditions such as active infectious diseases, ongoing malignancies, or active inflammatory or autoimmune disorders within the previous three months were also excluded.

### 2.3. Data Collection and Definitions:

We collected blood and urine samples prior to the initiation of SGLT2i and then at 3 months of follow-up. The following demographic and clinical data were recorded: age, sex, and diabetes status. The documented laboratory results included assessments of: eGFR, proteinuria, albuminuria, serum albumin, hemoglobin, glycemia, glycated hemoglobin (HbA1c), lipid profile, uric acid, and C-reactive protein (CRP).

Calculations of eGFR were performed using the Chronic Kidney Disease Epidemiology Collaboration 2009 (CKD-EPI-2009) formula (according to KDIGO 2024 Clinical Practice Guideline for the Evaluation and Management of Chronic Kidney Disease) [17].

We measured proteinuria and albuminuria using normalization to urinary creatinine, expressing them as urinary protein to creatinine ratio (UPCR mg/g) and urinary albumin to creatinine ratio (UACR mg/g). We categorized normoalbuminuric patients as having an albuminuria level of less than 30 mg/g. Values between 30 and 300 mg/g were defined as moderately increased albuminuria. Also, heavy albuminuria was characterized as the presence of more than 300 mg/g (according to current KDIGO guidelines) [17].

Urine samples were collected from eligible participants to quantify podocyte biomarkers, such as nephrin, podocin, and podocalyxin. Likewise, urinary podocyte bio-marker concentrations (expressed in µg/mg) were normalized to urinary creatinine to account for urine dilution, similar to standard UACR reporting.

Urinary biomarkers, as well as biochemical parameters, were quantified at 3 months follow-up. The difference between baseline and 3-month values was defined as Delta (∆) for each parameter (e.g., delta podocalyxin = baseline podocalyxin − podocalyxin at 3 months).

### 2.4. Laboratory Procedures

Urine samples were processed by centrifugation at 2600× *g* for 10 min. Aliquots were stored in 1.5 mL Protein LoBind tubes (Eppendorf, Hamburg, Germany) and subsequently preserved at −86 °C in an ultra-low temperature freezer (for a maximum time of 13 months).

The podocyte-derived biomarkers (nephrin, podocin, and podocalyxin) concentration in the urine was determined using commercially available ELISA kits, all from Antibodies Stockholm, Sweden (Human Nephrin ELISA Kit-A310801; Human NPHS2 ELISA Kit-A2577; Human PODXL ELISA Kit-A2580) according to the manufacturer’s protocol. Inter and intra-assay variability, the following minimum detection limits are reported: 0.04 ng/mL (for Nephrin), 0.24 ng/mL (for NPHS2), and 0.053 ng/mL (for Podocalyxin).

### 2.5. Statistical Analysis

Statistical analysis included both descriptive and inferential methods. Descriptive statistics were used to characterize the total group and the subgroups. Continuous variables were described using arithmetic means and standard deviations (or medians and interquartile ranges, as appropriate), while qualitative variables were described using frequencies and percentages. For consistency in UACR assessment, patients were categorized as normoalbuminuric (<30 mg/g) and albuminuric (≥30 mg/g), and analyses were performed accordingly.

Comparative analyses were conducted to evaluate differences between two subgroups, applying statistical tests depending on data distribution and variable type, such as Student test/Mann–Whitney test for quantitative variables and Chi-square test for qualitative variables. The distribution of quantitative variables was assessed using the Shapiro–Wilk test. Variables with a normal distribution were compared using Student’s *t*-test, whereas non-normally distributed variables were analyzed using the Mann–Whitney U test.

Associations between variables were explored using linear or non-linear correlation analyses. Additionally, multivariate linear regression models were performed to assess the relationships between independent variables and dependent variables.

Multicollinearity among independent variables was evaluated using the Variance Inflation Factor (VIF), and variables with VIF values greater than 5 were excluded from the multivariable model. After confirming that the regression assumptions were adequately satisfied, the remaining variables were entered into the final multivariable regression model. Regression coefficients (β) with corresponding 95% confidence intervals (95% CI) and *p*-values were reported.

A multiple linear regression analysis was performed to evaluate the associations between the independent variables and the dependent variable (ΔUACR, respectively, ΔeGFR). The assumptions of linear regression were assessed. The normality of residuals was evaluated through a histogram. Extreme cases were iteratively identified and excluded based on residual diagnostics until the distribution of residuals approximated normality. Homoscedasticity was assessed by inspection of plots of standardized residuals versus predicted values.

We used ROC (Receiver Operating Characteristic) curve analysis as a method to compare factors, to select an optimal cut-off value, and to assess the predictive performance of a binary classification. The optimal threshold was determined by maximizing Youden’s J index. We report the area under the ROC curve (AUC) and the 95% confidence interval (CI). The analysis was made using SPSS version 25.0 (IBM Corp., Armonk, NY, USA).

## 3. Results

Our cohort consisted of 88 patients. The main baseline characteristics of the participants are presented in Table 1.

The majority of patients were normoalbuminuric (57; 64.7%), while 15 (17.04%) had moderately increased albuminuria and 16 (18.2%) had severely increased albuminuria (UACR > 300 mg/g). A comparison between normoalbuminuric and albuminuric patients is provided in Table 2. Apart from albumin excretion-related parameters (UACR and albuminemia), normoalbuminuric patients were significantly older and exhibited lower baseline podocalyxin levels, with more stable podocalyxin values at 3 months.

Thirty-one patients were diabetic. There were more female patients in the diabetic group (80.6%), and they were slightly older (72 years old, 68.5–77). Moreover, they had lower HDL (49 mg/dL (40–56)) and higher triglycerides (147 mg/dL (93–196)). Podocin concentrations at baseline were significantly elevated in the diabetic group compared with the non-diabetic group. The other parameters were not different between the two groups. Complete comparison of diabetic and non-diabetic patients at baseline is presented in Supplementary Table S1.

**Figure 1:**
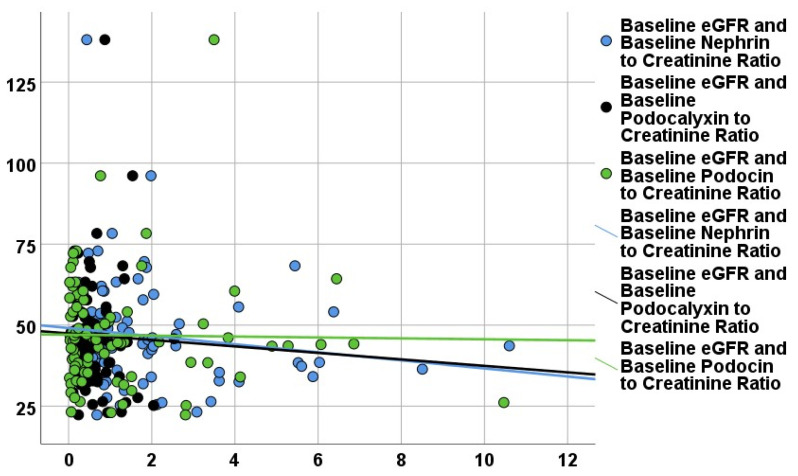
Correlation of urinary podocyte biomarkers with renal parameters at baseline. Relationship between urinary podocyte biomarkers and estimated glomerular filtration rate (eGFR).

**Figure 2:**
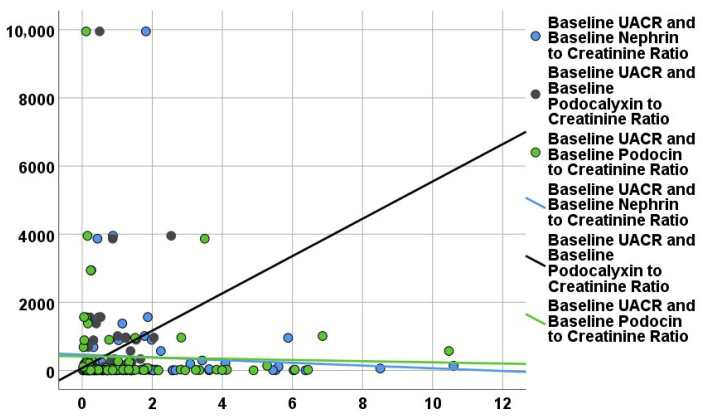
Correlation of urinary podocyte biomarkers with renal parameters at baseline. Relationship between urinary podocyte biomarkers and urinary albumin-to-creatinine ratio (UACR).

Correlation analysis was performed between baseline podocyte biomarkers, eGFR (Figure 1), and UACR (Figure 2), and other quantitative data at baseline.

At baseline, podocyte biomarkers were strongly correlated with each other and were associated with selected lipid metabolism parameters, whereas only podocalyxin demonstrated a significant association with UACR (Table 3).

Subgroup analyses of normoalbuminuric patients and albuminuric patients revealed correlation patterns similar to those observed in the overall cohort.

At three months, eGFR decreased (43.82 mL/min/1.73 m^2^ vs. 45.0 mL/min/1.73 m^2^), albeit the difference was not significant. UACR and UPCR remained stable (UACR 22.54 mg/g vs. 19.09 mg/g; *p* = 0.662; UPCR 160.03 mg/g vs. 117.55 mg/g; *p* = 0.06).

We assessed the correlations between changes in eGFR from baseline to 3 months (ΔeGFR), changes in UACR over the same period (ΔUACR), and the corresponding changes in podocyte biomarker levels (Table 4). Baseline podocalyxin, as well as Δpodocalyxin and Δpodocin, demonstrated the most consistent and statistically significant correlations with changes in renal function (ΔeGFR) and albuminuria (ΔUACR).

In the multivariate analysis with ΔeGFR and ΔUACR as dependent variables, Δpodocalyxin emerged as the only independent predictor of both ΔeGFR (Table 5) and ΔUACR (Table 6), while baseline podocalyxin significantly correlated with ΔUACR (Table 6). In the multivariate regression analysis, we included variables susceptible to predicting changes in eGFR and UACR, respectively. Baseline levels of podocyte biomarkers, as well as their changes during the 3-month follow-up period, were selected, as our study hypothesized that these biomarkers and their early dynamics may have predictive value for subsequent changes in eGFR and albumin excretion. Additionally, for multivariate analysis in which ΔUACR was the dependent variable, baseline eGFR and ΔeGFR were selected as variables of interest. This approach was chosen because the initial improvement in proteinuria is correlated with eGFR dip, and both are recognized markers of the therapeutic effect of SGLT2 inhibitors. Conversely, in the multivariate analysis with ΔeGFR as the dependent variable, both baseline values and ΔUACR/UPCR were initially introduced; however, ΔUACR/UPCR were subsequently excluded due to multicollinearity.

We compared patients exhibiting an increase versus a decrease value in podocyte biomarkers after three months of follow-up, focusing on eGFR and UACR dynamics. No significant differences in eGFR or UACR changes were noted with variations in urinary nephrin. In contrast, a decreased podocin was associated with a significant early eGFR dip, while a decreased podocalyxin was associated with both a significant eGFR dip and a reduction in UACR (Table 7).

We tried to evaluate whether baseline urinary podocyte biomarkers exhibit clinically meaningful cut-off values for predicting short-term changes in renal function and albuminuria.

**Figure 3:**
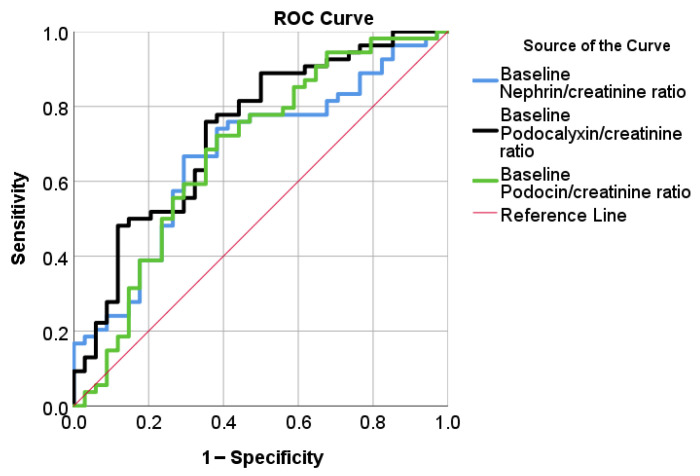
ROC curves evaluating the predictive performance of baseline urinary podocyte biomarkers for eGFR dynamics. Receiver operating characteristic (ROC) curves illustrating the discriminative ability of baseline urinary nephrin/creatinine, podocalyxin/creatinine, and podocin/creatinine ratios to predict changes in eGFR during follow-up. Sensitivity is plotted against 1 − specificity, with the diagonal reference line indicating no discriminative ability.

Patients were stratified according to early eGFR dynamics into dippers (eGFR decline; ΔeGFR ≥ 0) and non-dippers (no decline; ΔeGFR < 0). Biomarker-specific baseline cut-off values for nephrin, podocalyxin, and podocin were derived from ROC curve analyses (Figure 3) and used to evaluate their ability to discriminate between these groups. Baseline podocalyxin demonstrated the highest discriminative accuracy among the evaluated biomarkers.

The identified cut-off values for baseline nephrin/creatinine ratio were [AUC = 0.67, 242 95%CI (0.56, 0.79), *p* = 0.006] cut-off = 0.97 μg/mg; Baseline podocalyxin/creatinine ratio 243 [AUC = 0.73, 95%CI (0.63, 0.84), *p* < 0.001] cut-off = 0.36 μg/mg; baseline podocin/creatinine 244 ratio [AUC = 0.68, 95%CI (0.56, 0.80), *p* = 0.005] cut-off = 0.26 μg/mg.

**Figure 4:**
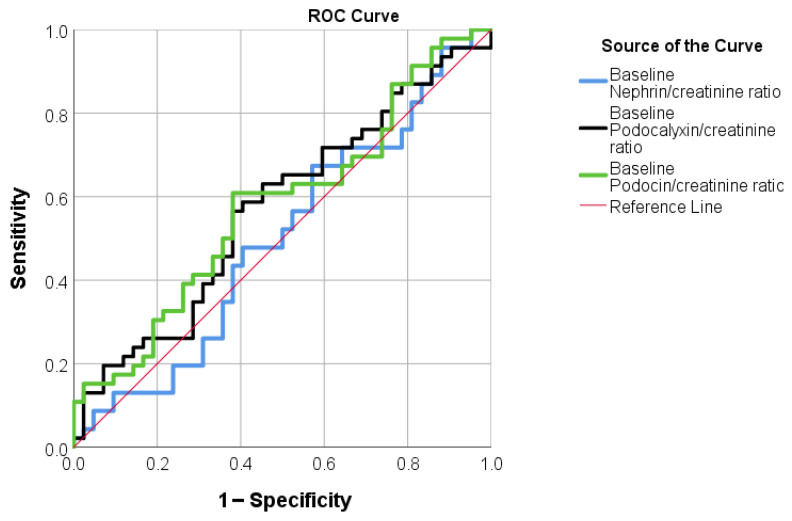
ROC curves evaluating the predictive performance of urinary podocyte biomarkers and UACR.

In contrast, when patients were stratified according to changes in UACR, no statistically significant discrimination was observed between groups at baseline; baseline Nephrin/creatinine ratio [AUC = 0.50, 95%CI (0.38, 0.62), *p* = 0.993]; baseline Podocalyxin/creatinine ratio [AUC = 0.57, 95%CI (0.45, 0.69), *p* = 0.249]; baseline Podocin/creatinine ratio [AUC = 0.58, 95%CI (0.46, 0.70), *p* = 0.213] (Figure 4).

## 4. Discussion

In light of the strong and compelling evidence demonstrating the efficacy of SGLT2i in slowing the progression of kidney disease, current 2024 KDIGO Guidelines for the Evaluations and Management of Chronic Kidney Disease established the use of this class of medication as a 1A recommendation for patients with diabetes and other various causes of CKD, when there is eGFR of >20 mL/min/1.73 m^2^ and UACR > 200 mg/g. Furthermore, there is also a 2b recommendation for the use of SGLT2i (eGFR 20 to 45 mL/min per 1.73 m^2^ with UACR < 200 mg/g). The lower strength of recommendation is derived from the fact that normoalbuminuric patients were underrepresented in the CKD clinical trials. Although approximately half of the EMPA-KIDNEY trial cohort had normal or moderately increased albuminuria, the number of events in this category of patients was low, and evidence for a benefit in slowing GFR decline in these subgroups is derived from a post hoc analysis [3,18].

Real-life experience accumulated at a high pace supports the beneficial effects of SGLT2i in normoalbuminuric patients. As such, in the Optimize CKD study, no significant differences in renal or cardiovascular endpoints were observed between patients with high UACR (>200 mg/g) and those with low UACR (<200 mg/g) [19]. Multiple studies have shown that SGLT2i therapy consistently attenuates eGFR decline and reduces albuminuria, conferring significant renal and cardiovascular benefits even in patients with low albuminuria levels (UACR < 200 mg/g), including non-diabetic and normoalbuminuric individuals [18,19,20].

Taken together, these data provide compelling evidence that SGLT2i confer clinically meaningful benefits even in patients with normoalbuminuria and microalbuminuria.

However, whereas a marked reduction in proteinuria (approximately 30–50%) is promptly observed in proteinuric patients following initiation of SGLT2i [2,3,21], and early antiproteinuric response has been correlated with improved long-term renal outcomes [9,10], this effect is less evident and more difficult to quantify in normoalbuminuric or microalbuminuric patients due to their low baseline UACR levels. Consequently, demonstration of the therapeutic benefit of SGLT2 inhibition in these populations requires large patient cohorts or meta-analytic evidence. At the individual patient level, short-term effects are difficult to ascertain; a prolonged follow-up period is often necessary to document stabilization of the eGFR slope.

This raises the question of whether there is a reliable biomarker that is rapidly modulated by SGLT2i therapy and whose early modification could predict long-term treatment effects, particularly in normoalbuminuric patients.

The main renal effect of SGLT2i is hemodynamic alleviation of hyperfiltration, through restoration of tubulo-glomerular feedback with afferent arteriole vasoconstriction and efferent arteriole vasodilation induced by second-degree mediators such as adenosine, prostaglandins, and reduced renin release [22,23,24]. However, mounting evidence suggests that SGLT2i exert pleiotropic effects beyond hemodynamic modulation, including anti-inflammatory and antifibrotic actions, reduction in oxidative stress and advanced glycation end-products (AGEs), improvements in anemia and tissue oxygen delivery, metabolic shift toward ketogenesis with enhanced intrarenal energy efficiency, and direct myocardial effects that improve cardiac contractility efficiency [25,26].

Another potentially relevant yet insufficiently explored mechanism is the attenuation of podocyte injury. Across multiple experimental models of diabetic nephropathy, treatment with SGLT2i has been associated with preservation of podocyte integrity, reflected by higher podocyte count and density, along with reduced podocyte foot process effacement [27,28]. Similar findings have been reported in experimental models of non-diabetic kidney disease, in which SGLT2 inhibition mitigated proteinuria, glomerular injury, and preserved podocyte structure and viability, limiting podocyte dysfunction and depletion [6].

Several mechanistic pathways that mediate SGLT2i-induced podocyte preservation have been implicated. In experimental diabetic models, SGLT2 inhibition attenuated oxidative stress and carbonyl activation [28,29,30], reduced AGE accumulation [27,31], and downregulated inflammatory mediators (e.g., IL-6, MCP-1) and profibrotic signaling (including TGF-β), with concomitant reductions in collagen synthesis and extracellular matrix deposition [27,29,31,32].

One of the most compelling insights from recent experimental work on SGLT2i is the demonstration that podocytes express SGLT2. Cassis et al. (2018) first reported increased podocyte SGLT2 expression in a mouse model of protein-overload proteinuria and confirmed constitutive SGLT2 expression in human podocytes in vitro, with upregulation following albumin exposure. The colocalization of SGLT2 with key podocyte cytoskeletal proteins, such as synaptopodin, in non-diabetic experimental models, including lupus nephritis [33] and Alport syndrome [34]—confirmed both in vivo and in cultured podocytes—provides strong evidence for podocyte-intrinsic SGLT2 expression. Moreover, SGLT2i have been linked to increased nephrin expression and stabilization [6]. These findings support the concept that SGLT2 inhibitors may exert direct podocyte-protective effects in proteinuric kidney disease [6].

Biomarkers of podocyte integrity, such as slit diaphragm proteins, such as/like nephrin [35], have long been assessed in both diabetic and non-diabetic kidney disease as early indicators of kidney injury. Nephrin is a key slit diaphragm transmembrane protein ensuring selective filtration and podocyte survival signaling [8,36]. Its levels increase early in the course of the disease, preceding the development of significant albuminuria, a finding subsequently corroborated by multiple clinical studies, largely conducted in diabetic cohorts [7]. Comparable evidence has also been reported for podocin [37]. Podocin is another pivotal slit diaphragm protein that stabilizes the complex by linking nephrin to the podocyte actin cytoskeleton, maintaining barrier integrity [8,36]. Another extensively studied podocyte biomarker is podocalyxin, which is a protein localized at the apical surface of podocytes. As a major surface glycoprotein, it contributes to charge-selective filtration by repelling anionic proteins and prevents excessive adhesion of podocyte foot processes, thereby preserving the characteristic architecture of the slit diaphragm [8,36,38]

The impact of SGLT2i on podocyte biomarkers has been approached in experimental animal models. In various experimental diabetic and non-diabetic settings, SGLT2i attenuated podocyte injury and loss, resulting in reduced glomerular damage and proteinuria [6]. Furthermore, Oraby et al. (2019) emphasized that podocyte protein injury may precede albuminuria and showed that, in a diabetic rat model, dapagliflozin restored nephrin expression and reduced oxidative stress, thereby preserving podocyte integrity and limiting glomerulosclerosis [29]. 

Clinical evidence on SGLT2i–associated modulation of podocyte injury biomarkers remains sparse. To our knowledge, only four small prospective studies and one retrospective study have addressed this issue in type 2 diabetes, with none including non-diabetic CKD patients. Therefore, we sought to evaluate the short-term effects of SGLT2i on podocyte biomarkers in SGLT2i-naïve patients and to analyze associations between baseline characteristics and 3-month changes in podocyte markers, eGFR, and UACR.

Our cohort included CKD patients, both diabetic and non-diabetic. As there were no significant differences between diabetic and non-diabetic patients from the point of view of kidney disease (eGFR and albuminuria), we did not analyze them separately.

Baseline kidney function was highly variable, with eGFR spanning a wide range from stage IV CKD to normal values, and UACR ranging from normoalbuminuria to nephrotic-range proteinuria.

At baseline, podocyte biomarkers demonstrated a moderate magnitude. This can be expected from a pathogenic perspective since they all reflect podocyte injury. However, their association with baseline UACR was weak, reaching statistical significance only for podocalyxin, with a moderate correlation coefficient (r = 0.3). No statistically significant association was observed between these biomarkers and baseline eGFR.

When analyzing changes in podocyte biomarkers at 3 months, it was observed that baseline podocalyxin, as well as changes in podocalyxin (Δpodocalyxin) and podocin (Δpodocin), were positively correlated with changes in both eGFR (ΔeGFR) and UACR (ΔUACR). Thus, a decrease in these podocyte biomarkers, especially podocalyxin, paralleled an albuminuria improvement and a more significant early decrease in eGFR, consistent with the expected hemodynamic dip induced by SGLT2i therapy. These associations remained significant despite only modest changes in eGFR and UACR at 3 months, which were not statistically significant in our cohort. It is true that the strength of these associations, although statistically significant, was moderate in magnitude, with correlation coefficients not exceeding 0.38. As such, these results suggest that urinary podocalyxin and podocin may represent biomarkers for detecting early renal responses to SGLT2i therapy, although their predictive power appears to be moderate.

We also assessed whether baseline urinary podocyte biomarkers could segregate patients who experienced an early eGFR decline (“dippers”) from those without an eGFR decline. ROC curve analyses yielded significant cut-off values for baseline nephrin, podocalyxin, and podocin, indicating all three biomarkers were able to predict short-term eGFR dipping. However, the AUC values for the three biomarkers indicated moderate predictive accuracy, ranging from 0.56 to 0.73, with the highest value observed for podocalyxin. This suggests that podocalyxin may have moderate predictive value for identifying patients who develop an early eGFR dip. In contrast, no baseline podocyte biomarker provided a significant cut-off for predicting short-term UACR dynamics.

In our cohort, urinary podocalyxin was the strongest predictor of early SGLT2i response, followed by podocin, whereas nephrin (baseline and Δnephrin) did not predict changes in UACR or eGFR. A plausible explanation relates to the temporal dynamics of nephrin excretion, which increases early at disease onset, followed by a decline as disease progresses. Consistent with this dynamic, Fukuda et al. (2012) demonstrated in an experimental podocyte injury model that podocin expression remains persistently elevated, whereas nephrin exhibits an early peak followed by a decline, leading to the proposal of the podocin/nephrin ratio as a more robust indicator of sustained podocyte damage [37]. In line with these observations, a meta-analysis by Mesfine et al. (2024) reported higher diagnostic performance of nephrin in acute kidney injury settings (e.g., preeclampsia) than in diabetic kidney disease [39]. In contrast, given its apical transmembrane localization, podocalyxin may be more readily shed into the urine, potentially accounting for its higher sensitivity as a podocyte biomarker in our study.

Our findings are consistent with previously published clinical studies that demonstrated the effect of SGLT2i on modulating podocyte injury. The first one, conducted by Tian et al. (2022), investigated the effects of SGLT2 inhibition on podocyte injury and renal fibrosis in type 2 diabetes. In 68 patients followed for 3 months, urinary nephrin—used as a marker of podocyte damage—was reduced with SGLT2i therapy, and nephrin levels were positively associated with albuminuria [12]. This contrasts with our findings, as baseline nephrin was not significantly associated with baseline albuminuria or with early changes in albuminuria and eGFR. One possible explanation for the difference between our cohort and that of Tian et al. is the inclusion of non-diabetic patients and the high proportion of normoalbuminuric individuals in our cohort (57/88), whereas Tian’s study included only diabetic patients with more advanced kidney disease. Notably, the most pronounced decrease in nephrin in Tian’s research was observed in proteinuric patients, while the change was not significant in normoalbuminuric patients. Durcan et al. (2022) studied 40 male patients with type 2 diabetes, divided into 2 groups—SGLT2i vs. control—over 6 months and assessed urinary podocalyxin and synaptopodin as podocyte injury markers. Both biomarkers were markedly reduced in the SGLT2i group at the end of the follow-up period, consistent with preservation of podocyte structural integrity [14]. Similarly, in our study, the decrease in podocalyxin was associated with improvement in albuminuria and the early eGFR dip, suggesting an effect related to SGLT2i therapy. There is also a small randomized controlled trial (RCT) in diabetic patients conducted by Shi et al. (2025), which included 78 patients with early type 2 diabetic nephropathy (T2DN), assigning 39 to dapagliflozin for 3 months. Serum podocyte injury markers (podocalyxin and nephrin) increased significantly in the treated group, suggesting improved podocyte integrity [11]. The difference compared with our study is the assessment of these biomarkers in serum, whereas the majority of studies, including ours, have evaluated urinary values. While increased synthesis and expression of nephrin and podocalyxin might result in higher serum levels, a more direct reflection of improvement in podocyte biology is their decreased shedding in the urine. To the best of our knowledge, there are no clinical studies in medical literature that have evaluated both serum and urine levels of nephrin in order to assess the effect of SGLT2i treatment in CKD patients. Other reports are more reserved concerning the value of podocyte biomarkers for predicting kidney outcomes after SGLT2i: a 2026 retrospective study by Nugroho et al. in 119 patients with type 2 diabetes showed that baseline urinary nephrin and podocin can reflect early glomerular injury but have limited predictive value for 12-month renal function or albuminuria progression [15]. Li et al. (2025) compared 24 patients with diabetic kidney disease receiving SGLT2i with 25 controls and quantified urinary podocin, podocalyxin, and synaptopodin via urinary mRNA levels. Biomarkers remained unchanged after 3 months of therapy but were significantly lower than in controls, with no significant correlation with the estimated glomerular filtration rate (eGFR) or albuminuria at baseline or follow-up [13].

To our knowledge, this is the first study to include non-diabetic patients and a substantial proportion of normo- and microalbuminuric subjects, thereby adding to the knowledge gap in this area.

However, this study has several limitations. The relatively small sample size limits statistical power and may increase the risk of a type II error. In addition, podocyte biomarkers were evaluated only over a 3-month period; therefore, longer prospective follow-up is required to confirm whether the early dynamics of these biomarkers have long-term predictive value for kidney outcomes. Furthermore, the observational design of the study allows identification of associations between podocyte biomarkers and the dynamics of eGFR and UACR, but it does not establish a causal relationship. Nevertheless, such a relationship is biologically plausible based on the current understanding of podocyte biology.

## 5. Conclusions

Our findings support the concept that SGLT2i exert pleiotropic renoprotective effects, including attenuation of early podocyte injury. In this context, our data suggest that urinary podocyte biomarkers, especially podocalyxin—and to a lesser extent podocin—may serve as sensitive indicators of early renal response to SGLT2i therapy, particularly in normoalbuminuric CKD.

## Figures and Tables

**Table 1:** General baseline characteristics of patients.

Variable	Value
	N = 88
Age (years)	70.5 (66; 75.5)
Male n (%)	33 (37.5)
Diabetes n (%)	31 (36)
eGFR (mL/min/1.73 m^2^)	45 (36.85; 53.15)
UPCR (mg/g)	117.55 (79.28; 323.06)
UACR (mg/g)	19.09 (6.28; 164.93)
Albumin (g/dL)	4.23 (4.03; 4.45)
Hemoglobin (g/dL)	12.9 (12; 14)
HbA1c (%)	6.44 (5.97; 7.38)
Total cholesterol (mg/dL)	164 (138; 192)
LDL-cholesterol (mg/dL)	90 (69.27; 123)
HDL-cholesterol (mg/dL)	51.05 (44; 59.5)
Triglycerides (mg/dL)	111.29 (85.5; 151.5)
Uric acid (mg/dL)	6.8 (5.98; 8.16)
CRP (mg/dL)	0.25 (0.13; 0.56)
Nephrin/creatinine ratio (µg/mg)	1.04 (0.7; 2.01)
Podocalyxin/creatinine ratio (µg/mg)	0.45 (0.25; 0.83)
Podocin/creatinine ratio (µg/mg)	0.44 (0.14; 1.33)

eGFR, estimated glomerular filtration rate; UPCR, urinary protein/creatinine ratio; UACR, urinary albumin/creatinine ratio; HbA1c, glycated hemoglobin; LDL, low-density lipoprotein; HDL, high-density lipoprotein; CRP, C-reactive protein.

**Table 2:** Comparison of patient characteristics across albuminuria categories.

Variables	UACR < 30 mg/g (n = 57)	UACR ≥ 30 mg/g (n = 31)	*p*-Value
Male patients n (%)	20 (35.1)	13 (41.9)	0.526
Age (years)	72 (68; 77)	67 (54; 71)	**0.001**
Diabetes mellitus n (%)	23 (41.1)	8 (26.7)	0.185
Baseline eGFR (mL/min/1.73 m^2^)	45.5 (38.4; 51.2)	44.8 (35.9; 61.05)	0.986
ΔeGFR mL/min/1.73 m^2^	2.14 (−4.12; 5.71)	1.13 (−1.7; 5.77)	0.558
Baseline nephrin/creatinine ratio (µg/mg)	0.94 (0.7; 1.99)	1.24 (0.87; 2.66)	0.220
Δnephrin/creatinine ratio (µg/mg)	−0.48 (−1.54; 0.12)	−0.12 (−1.15; 1)	0.177
Baseline podocalyxin/creatinine ratio (µg/mg)	0.38 (0.21; 0.58)	0.69 (0.36; 1.24)	**0.001**
Δpodocalyxin/creatinine ratio (µg/mg)	−0.1 (−0.41; 0.05)	−0.05 (−0.3; 0.47)	**0.045**
Baseline podocin/creatinine ratio (µg/mg)	0.47 (0.17; 1.33)	0.28 (0.12; 1.18)	0.419
Δpodocin/creatinine ratio (µg/mg)	−0.17 (−1.41; 0.28)	−0.04 (−0.21; 0.41)	0.065
Baseline UPCR mg/g	90.33 (68.64; 117.55)	550.81 (261.4; 1391)	**<0.001**
ΔUPCR (mg/g)	−32.67 (−77.09; −5.05)	60.67 (−113.69; 580.72)	**0.044**
Serum albumin (g/dL)	4.3 (4.13; 4.45)	4.06 (3.81; 4.34)	**0.011**
Δserum albumin (g/dL)	0.01 (−0.15; 0.13)	−0.11 (−0.27; 0.08)	0.219
Hemoglobin (g/dL)	12.7 (12; 13.9)	13.4 (11.85; 14.4)	0.334
Δhemoglobin (g/dL)	−0.3 (−0.85; 0.3)	−0.45 (−1.1; 0)	0.215
HbA1c (%)	6.76 ± 1.07	6.81 ± 1.49	0.945
ΔHbA1c (%)	0.41 ± 0.49	0.37 ± 1.19	0.923
Total cholesterol (mg/dL)	164 (141; 188)	163 (130; 231)	0.937
**Δ**total cholesterol (mg/dL)	9 (−13.5; 28.5)	9 (−23.5; 29)	0.785
LDL-cholesterol (mg/dL)	88 (70; 110)	96 (65; 155)	0.292
**Δ**LDL-cholesterol (mg/dL)	8 (−5.5; 25.65)	4 (−15.5; 24)	0.359
HDL-cholesterol (mg/dL)	51.05 (44.65; 60)	51.5 (39; 56.85)	0.417
**Δ**HDL-cholesterol (mg/dL)	−0.07 ± 9.36	−1.76 ± 11.46	0.720
Triglycerides (mg/dL)	111.57 (85.5; 153.5)	109 (93; 148)	0.738
Δtriglycerides (mg/dL)	3 (−19.5; 26)	1.4 (−20.5; 19)	0.629
Uric acid (mg/dL)	6 (5; 7)	6 (5; 7.5)	0.538
Δuric acid (mg/dL)	15.34 (−0.22; 26.92)	10.24 (5.56; 19.16)	0.388
CRP (mg/dL)	0.26 (0.13; 0.56)	0.24 (0.13; 0.48)	0.703
ΔCRP (mg/dL)	0 (−0.13; 0.1)	−0.02 (−0.18; 0.12)	0.639

eGFR, estimated glomerular filtration rate; UPCR, urinary protein/creatinine ratio; UACR, urinary albumin/creatinine ratio; HbA1c, glycated hemoglobin; LDL, low-density lipoprotein; HDL, high-density lipoprotein; CRP, C-reactive protein.

**Table 3:** Correlations between urinary podocyte biomarkers, renal parameters, and other biochemical markers at baseline in the total group.

Parameters	Baseline Nephrin/Creatinine Ratio (n = 88)		Baseline Podocalyxin/Creatinine Ratio (n = 88)		Baseline Podocin/Creatinine Ratio (n = 88)	
	r	*p*	r	*p*	r	*p*
Nephrin/creatinine ratio (µg/mg)		-	0.69	**<0.001**	0.36	**0.001**
Podocalyxin/creatinine ratio (µg/mg)	0.69	**<0.001**	-		0.52	**<0.001**
Podocin/creatinine ratio (µg/mg)	0.36	**0.001**	0.52	**<0.001**	-	
eGFR (mL/min/1.73 m^2^)	−0.19	0.071	−0.12	0.253	−0.11	0.318
UPCR (mg/g)	0.13	0.228	0.36	**0.001**	0.11	0.290
UACR (mg/g)	−0.06	0.580	0.3	**0.005**	−0.03	0.810
Total cholesterol (mg/dL)	0.23	**0.038**	0.21	0.056	0.16	0.144
LDL-cholesterol (mg/dL)	0.28	**0.008**	0.24	**0.027**	0.14	0.195
HDL-cholesterol (mg/dL)	0.27	**0.012**	0.26	**0.017**	0.23	**0.032**
Triglycerides (mg/dL)	−0.05	0.630	0.02	0.832	−0.16	0.166

r—coefficient of correlation; eGFR, estimated glomerular filtration rate; UPCR, urinary protein/creatinine ratio; UACR, urinary albumin/creatinine ratio; LDL, low-density lipoprotein; HDL, high-density lipoprotein.

**Table 4:** Correlations between changes in eGFR, UACR, and urinary podocyte biomarkers (baseline to 3 months, Δ) in the total group.

	ΔeGFR		ΔUACR	
	Coefficient of Correlation	*p*	Coefficient of Correlation	*p*
Baseline nephrin/creatinine ratio (µg/mg)	0.28	0.09	0.05	0.17
Baseline podocin/creatinine ratio (µg/mg)	0.30	0.05	0.08	0.432
Baseline podocalyxin/creatinine ratio (µg/mg)	**0.39**	**0.001**	**0.38**	**0.001**
Δnephrin/creatinine ratio (µg/mg)	0.18	0.09	0.06	0.50
Δpodocin/creatinine ratio (µg/mg)	**0.23**	**0.009**	**0.23**	**0.035**
Δpodocalyxin/creatinine ratio (µg/mg)	**0.31**	**0.04**	**0.38**	**0.001**

eGFR, estimated glomerular filtration rate; UACR, urinary albumin/creatinine ratio.

**Table 5:** Multivariable linear regression analysis of eGFR changes (ΔeGFR) in relation to urinary podocyte biomarkers.

	Unstandardized Coefficients			95.0% Confidence Interval for B	
	B	Standard Error	*p*	Lower Bound	Upper Bound
(Constant)	0.310	1.223	0.801	−2.128	2.748
Baseline UPCR (mg/g)	0.000	0.001	0.618	−0.002	0.001
Baseline UACR (mg/g)	0.000	0.001	0.797	−0.001	0.001
Baseline nephrin/creatinine ratio (µg/mg)	−0.245	0.425	0.566	−1.091	0.601
Δnephrin/creatinine ratio (µg/mg)	0.086	0.341	0.800	−0.593	0.766
Baseline podocalyxin/creatinine ratio (µg/mg)	3.536	1.929	0.071	−0.308	7.380
Δpodocalyxin/creatinine ratio (µg/mg)	2.952	1.430	**0.042**	0.103	5.801
Baseline podocin/creatinine ratio (µg/mg)	0.539	0.405	0.188	−0.269	1.347
Δpodocin/creatinine ratio (µg/mg)	−0.311	0.182	0.093	−0.675	0.053

eGFR, estimated glomerular filtration rate; UACR, urinary albumin/creatinine ratio.

**Table 6:** Multivariable linear regression analysis of UACR changes (ΔUACR) in relation to urinary podocyte biomarkers.

Model	Unstandardized Coefficients		Standardized Coefficients			95.0% Confidence Interval for B	
	B	Std. Error	Beta	t	Sig.	Lower Bound	Upper Bound
(Constant)	−151.064	144.994		−1.042	0.301	−439.907	137.779
Baseline nephrin/creatinine ratio (µg/mg)	−35.870	22.202	−0.186	−1.616	0.110	−80.099	8.359
Δnephrin/creatinine ratio (µg/mg)	−24.729	18.766	−0.160	−1.318	0.192	−62.113	12.655
Baseline podocalyxin/creatinine ratio (µg/mg)	401.779	102.769	0.512	3.910	**0.000**	197.053	606.504
Δpodocalyxin/creatinine ratio (µg/mg)	169.344	78.462	0.260	2.158	**0.034**	13.040	325.647
Baseline podocin/creatinine ratio (µg/mg)	−5.701	20.968	−0.029	−0.272	0.786	−47.471	36.068
Δpodocin/creatinine ratio (µg/mg)	4.809	9.549	0.050	0.504	0.616	−14.214	23.831
ΔeGFR (mL/min/1.73 m^2^)	0.388	4.326	0.010	0.090	0.929	−8.230	9.005
Baseline eGFR (mL/min/1.73 m^2^)	2.419	2.699	0.088	0.896	0.373	−2.958	7.796
ΔUPCR (mg/g)	0.250	0.094	0.543	2.664	**0.009**	0.063	0.436
Baseline UPCR (mg/g)	−0.251	0.062	−0.840	−4.022	**0.000**	−0.376	−0.127

eGFR, estimated glomerular filtration rate; UACR, urinary albumin/creatinine ratio.

**Table 7:** Changes in eGFR and UACR stratified by urinary podocyte markers dynamics.

Δnephrin/creatinine (µg/mg)			
	<0 (n = 57)	>=≥0 (n = 31)	***p***
ΔeGFR	−0.65 ± 9.95	2.44 ± 6.45	0.122
ΔUACR mg/g	0.18 (−9.97; 14.59)	1.17 (−12.4; 17.6)	0.141
Δpodocalyxin/creatinine (µg/mg)			
	<0 (n = 56)	≥0 (n = 32)	*p*
ΔeGFR	−1.07 ± 8.37	3.07 ± 9.47	**0.036**
ΔUACR mg/g	−2.13 (−12.32; 8.82)	6.47 (−3.25; 83.51)	**0.044**
Δpodocin/creatinine (µg/mg)			
	<0 (n = 56)	≥0 (n = 32)	*p*
ΔeGFR	−0.45 (−4.82; 5.16)	4.15 (0.59; 6.63)	**0.002**
ΔUACR mg/g	−1.52 (−11.89; 10.27)	6.26 (−11.6; 23.93)	0.311

eGFR, estimated glomerular filtration rate; UACR, urinary albumin/creatinine ratio.

## Data Availability

The datasets analyzed during the current study are not publicly available due to ethical and privacy restrictions, as they contain anonymized patient data. However, the data are available from the corresponding author upon reasonable request and with permission from the relevant institutional ethics committee.

## Supplementary Materials

The following supporting information can be downloaded at: https://www.mdpi.com/article/10.3390/life16030529/s1. Table S1: Comparison of clinical and laboratory parameters according to diabetes status.

## Abbreviations

The following abbreviations are used in this manuscript:

SGLT2i	Sodium–glucose cotransporter–2 inhibitors
CKD	Chronic kidney disease
eGFR	Estimated glomerular filtration rate
HbA1c	Glycated hemoglobin
CRP	C-reactive protein
UPCR	urinary protein to creatinine ratio
UACR	urinary albumin to creatinine ratio
LDL	low-density lipoprotein
HDL	high-density lipoprotein
ROC	Receiver operating characteristics
AUC	Area under the curve
