# Supplementary material for: Dynamics and Predictive Values of Urinary Podocyte Biomarkers Following SGLT2 Inhibition in CKD
Source: Life (Basel). 2026 Mar 23;16(3):529. doi: 10.3390/life16030529 (PMC13027516; doi:10.3390/life16030529)
Supplement: Supplementary file 1 [file life-16-00529-s001.zip › life-4204407-supplementary.pdf]

Supplementary Table S1 - Comparison of clinical and laboratory parameters according to diabetes status

	**Without diabetes (n = 55)**	**With diabetes (n = 31)**	**p-value**
Male sex, n (%)	27 (49.1)	6 (19.4)	**0.006**
Age (years)	69 (63–73)	72 (68.5–77)	**0.049**
Baseline eGFR (mL/min/1.73 m²)	44.4 (38.4–49.7)	45.5 (36.25–56.5)	0.529
Δ eGFR	1.13 (−3.22–5.77)	1.66 (−4.13–5.33)	0.791
Total cholesterol (mg/dL)	166 (137–200)	164 (141–187)	0.767
Δ total cholesterol (mg/dL)	9 (−13.5–29)	9 (−19–29)	0.535
LDL-cholesterol (mg/dL)	95.5 (70–126)	88 (68.5–119.5)	0.529
Δ LDL-cholesterol (mg/dL)	7.5 (−6.38–31.5)	4 (−11–17)	0.165
HDL-cholesterol (mg/dL)	53.5 (45.5–63.25)	49 (40–56)	**0.039**
HDL-cholesterol at 3 months (mg/dL)	55 (47–60.5)	50 (40–57)	**0.023**
Δ HDL-cholesterol (mg/dL)	−0.5 ± 11.21	−0.81 ± 8.03	0.898
Triglycerides (mg/dL)	101.5 (81.5–125.5)	147 (93–196)	**0.003**
Triglycerides at 3 months (mg/dL)	91 (68–132)	147 (112–169)	**0.001**
Δ triglycerides (mg/dL)	5 (−15.5–25.5)	−1 (−21–31)	0.689
Baseline UPCR (mg/g)	106.69 (79.89–323.06)	117.55 (70.15–270.86)	0.968
Δ UPCR (mg/g)	−22.26 (−94.91–24.40)	−27.47 (−77.56–31.61)	0.996
Baseline UACR (mg/g)	19.95 (5.93–203.62)	16.38 (6.23–41.18)	0.301
Δ UACR (mg/g)	1.78 (−10.75–15.70)	0.18 (−11.97–11.63)	0.381
Baseline nephrin/creatinine ratio (µg/mg)	0.94 (0.69–1.99)	1.16 (0.73–1.98)	0.709
Δ nephrin/creatinine ratio (µg/mg)	−0.21 (−1.74–0.15)	−0.10 (−0.95–0.49)	0.271
Baseline podocalyxin/creatinine ratio	0.40 (0.26–0.69)	0.50 (0.25–0.89)	0.475
Δ podocalyxin/creatinine ratio	−0.10 (−0.41–0.10)	−0.05 (−0.38–0.20)	0.656
Baseline podocin/creatinine ratio	0.23 (0.11–1.21)	0.72 (0.33–1.38)	**0.034**
Δ podocin/creatinine ratio	−0.08 (−0.60–0.21)	−0.09 (−1.18–0.48)	0.847
